# Structural Brain Network Abnormalities and the Probability of Seizure Recurrence After Epilepsy Surgery

**DOI:** 10.1212/WNL.0000000000011315

**Published:** 2021-02-02

**Authors:** Nishant Sinha, Yujiang Wang, Nádia Moreira da Silva, Anna Miserocchi, Andrew W. McEvoy, Jane de Tisi, Sjoerd B. Vos, Gavin P. Winston, John S. Duncan, Peter N. Taylor

**Affiliations:** From the Translational and Clinical Research Institute (N.S.), Faculty of Medical Sciences, and Computational Neuroscience, Neurology, and Psychiatry Lab (N.S., Y.W., N.M.d.S., P.N.T.), Interdisciplinary Computing and Complex BioSystems Group, School of Computing, Newcastle University, Newcastle Upon Tyne; NIHR University College London Hospitals Biomedical Research Centre (Y.W., A.M., A.W.M., J.d.T., S.B.V., G.P.W., J.S.D., P.N.T.), UCL Institute of Neurology, Queen Square; Centre for Medical Image Computing (S.B.V.), University College London; Epilepsy Society MRI Unit (S.B.V., G.P.W., J.S.D), Chalfont St Peter, UK; and Department of Medicine (G.P.W.,), Division of Neurology, Queen's University, Kingston, Ontario, Canada.

## Abstract

**Objective:**

We assessed preoperative structural brain networks and clinical characteristics of patients with drug-resistant temporal lobe epilepsy (TLE) to identify correlates of postsurgical seizure recurrences.

**Methods:**

We examined data from 51 patients with TLE who underwent anterior temporal lobe resection (ATLR) and 29 healthy controls. For each patient, using the preoperative structural, diffusion, and postoperative structural MRI, we generated 2 networks: presurgery network and surgically spared network. Standardizing these networks with respect to controls, we determined the number of abnormal nodes before surgery and expected to be spared by surgery. We incorporated these 2 abnormality measures and 13 commonly acquired clinical data from each patient into a robust machine learning framework to estimate patient-specific chances of seizures persisting after surgery.

**Results:**

Patients with more abnormal nodes had a lower chance of complete seizure freedom at 1 year and, even if seizure-free at 1 year, were more likely to relapse within 5 years. The number of abnormal nodes was greater and their locations more widespread in the surgically spared networks of patients with poor outcome than in patients with good outcome. We achieved an area under the curve of 0.84 ± 0.06 and specificity of 0.89 ± 0.09 in predicting unsuccessful seizure outcomes (International League Against Epilepsy [ILAE] 3–5) as opposed to complete seizure freedom (ILAE 1) at 1 year. Moreover, the model-predicted likelihood of seizure relapse was significantly correlated with the grade of surgical outcome at year 1 and associated with relapses up to 5 years after surgery.

**Conclusion:**

Node abnormality offers a personalized, noninvasive marker that can be combined with clinical data to better estimate the chances of seizure freedom at 1 year and subsequent relapse up to 5 years after ATLR.

**Classification of Evidence:**

This study provides Class II evidence that node abnormality predicts postsurgical seizure recurrence.

Epilepsy surgery is an effective treatment for bringing seizure remission in drug-resistant focal epilepsies. However, it is underused.^1–3^ One reason for the underreferral of patients is the reservations regarding the uncertainty of its outcome.^3,4^ In ≈30% to 40% of individuals, seizures continue despite surgery, and the multidisciplinary team is unable to accurately predict this risk.^5–9^ Therefore, to better inform this clinical decision-making, there is a need to predict both seizure outcomes in the short term and the likelihood of seizure relapse in the long term.^8,10^

The incomplete removal of a wider epileptogenic network is increasingly being recognized as one of the reasons for continued seizures after surgery.^11,12^ Many studies, driven by the aforementioned hypothesis, have attempted predicting seizure outcomes from presurgical data.^9,13–20^ Most studies, however, have investigated brain networks without incorporating the knowledge of the planned/performed surgery into the analysis. Naturally, the outcome of epilepsy surgery will depend not only on the presurgery brain network but also on how the surgery (i.e., its location and extent) will affect the brain network.^21^ Including surgical data allows the inference of a surgically spared network, the subnetwork for which none of the connections are altered by surgery and are therefore expected to remain after the surgery. Thus, the presence of epileptogenic structures in the surgically spared network, a likely cause for seizure recurrence, needs investigation.

Studies using quantitative imaging have consistently demonstrated that in TLE there are structural abnormalities that involve brain structures beyond the hippocampus and the temporal lobe.^22–27^ Accumulating evidence suggests that these abnormalities configure a network of abnormal structures that may be involved in the generation of seizures.^11,28–31^ Indeed, the pathophysiologic mechanisms associated with epileptogenesis have a strong basis in aberrant neural connectivity.^32,33^ Therefore, quantifying the abnormalities before and expected to remain after surgery may inform postoperative seizure outcome.

The main goal of our study was to understand how structural network abnormalities relate to seizure outcomes after temporal lobe epilepsy (TLE) surgery. We investigated the abnormality of the surgically spared networks because, at a conceptual level, postoperative outcomes will likely be determined by what remains after surgery. Our study addresses 3 main questions. (1) Do patients with more abnormalities in the surgically spared network have worse postoperative seizure outcomes? (2) Does surgery reduce node (region) abnormality more in seizure-free patients than in patients who are not seizure-free? (3) If the node abnormality measure is to be used alongside common clinical variables of a patient, would it generalize to make patient-specific predictions on the chances of seizure freedom after surgery? Our study shows that the node abnormality in the surgically spared network is an important measure to be considered alongside other presurgical clinical factors to evaluate the risk of poorer seizure outcomes in patients with refractory TLE.

## Methods

### Participants

We studied 51 patients who underwent unilateral anterior temporal lobe resection at the National Hospital of Neurology and Neurosurgery, London, UK. Patients were followed up after surgery and classified according to the International League Against Epilepsy (ILAE) scale of seizure outcome at 12-month intervals.^34^ One year after the surgery, 34 patients were completely seizure-free (ILAE 1), 8 patients continued to have auras only (ILAE 2), and 9 patients were not seizure-free (ILAE 3–5). No patient had an outcome of ILAE 6.

ILAE surgical outcomes of seizure freedom were recorded annually at years 1 and 2 for all 51 patients, at year 3 for 45 patients, at year 4 for 37 patients, and at year 5 for 31 patients. We considered that a patient had a seizure relapse if, at any given year after year 1, the ILAE outcome of the patient changed from ILAE 1 to 2 to ILAE 3 to 5. If the ILAE outcome of a patient did not change to ILAE 3 to 5 and the follow-up duration was <5 years, it cannot be ascertained if the patient would have relapsed on a full 5-year follow-up. Therefore, beyond the known follow-up period, we did not include such patients in our analysis. On the basis of this criterion, among the year 1 patients at ILAE 1 to 2, the number of patients who relapsed (did not relapse) by the end of each subsequent years was as follows: 3 (39) by year 2; 8 (29) by year 3; 11 (21) by year 4; 13 (14) by year 5.

Notably, seizure outcome and seizure relapse are 2 different measures. Seizure outcome at year 1 categorized all 51 patients according to their ILAE score at 1 year after surgery. Seizure relapse categorized only those patients who were initially free from disabling seizures, i.e., ILAE 1 or 2, but then later at 5 years had a seizure reoccurrence. In labeling seizure relapse, we excluded the 9 patients who had seizure recurrence within 1 year after the surgery (i.e., ILAE 3–5 at year 1) to avoid any bias. The full patient details are provided in table S1, doi.org/10.5061/dryad.vx0k6djnv, and summarized in table 1.

**Table 1:**
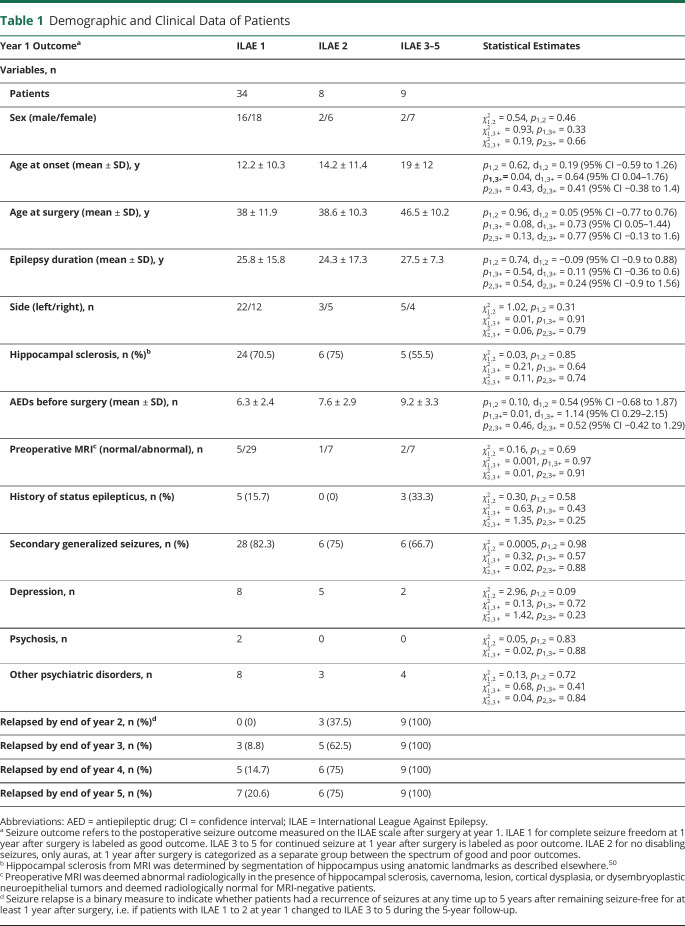
Demographic and Clinical Data of Patients

We also studied 29 healthy individuals, age and sex matched to patient group, with no significant medical history of neurologic or psychiatric problems.

### Standard Protocol Approvals, Registrations, and Patient Consents

The study was approved by the National Hospital for Neurology and Neurosurgery and the Institute of Neurology Joint Research Ethics Committee, and written informed consent was obtained from all participants. Data were analyzed in this study under the approval of the Newcastle University Ethics Committee (reference 1804/2020).

### MRI Acquisition, Data Processing, and Surgery Network

**Figure 1:**
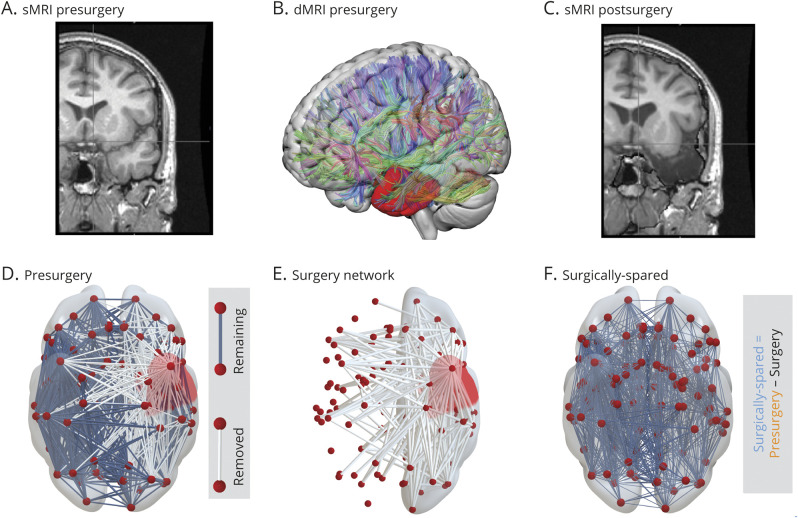
Estimating Patient-Specific Surgery Network Preoperative T1-weighterd (T1w) MRI of an example patient (A) and postoperative T1w MRI (C) were used to delineate the tissue resected by surgery. Resected tissue shown by the red resection mask in panel (B) was used with the preoperative diffusion MRI to infer brain networks. Presurgery network inferred from the number of streamlines connecting different regions of interest in panel (D) ignores the surgery information by not taking the resection mask into consideration. (E) Patient-specific surgery network showing the connections that were affected by the surgery. (F) Surgically spared network. dMRI = diffusion-weighted MRI; sMRI = structural MRI.

For each participant, T1-weighted structural MRI (sMRI) and diffusion-weighted MRI (dMRI) data were acquired before surgery. Within 1 year after surgery, T1-weighted sMRI data were acquired again for patients. As in our previous study,^21^ we used the postoperative sMRI to manually draw the resection mask in preoperative sMRI space, hence delineating the resected tissue. We parcellated the preoperative T1 image into 114 cortical and subcortical regions of interest (ROIs) derived from the predefined Geodesic Information Flow atlas and separately in 82 ROIs using the FreeSurfer Desikan-Killiany atlas in the native space of each participant. Along with streamlines inferred with dMRI tractography and regions inferred with parcellation, we incorporated the information of resected tissue to infer 2 networks: presurgery network and surgically spared network. The presurgery streamline network is the whole-brain network depicting the number of streamlines connecting predefined parcellated ROIs. The surgically spared network is a subnetwork of the presurgery network that is inferred after removal of the streamlines that intersected the resection mask. By definition, surgery can cause only an immediate reduction in the number of streamlines. Therefore, we specified that the surgically spared network contains only those network edges that are not expected to change in streamline count after surgery (i.e., edges where their streamlines do not pass through/into the resection cavity). The overall concepts are illustrated in figure 1. Detailed imaging protocols and data processing pipeline to infer these networks are described fully in supplementary Methods, doi.org/10.5061/dryad.vx0k6djnv.

### Node Abnormality Computation

**Figure 2:**
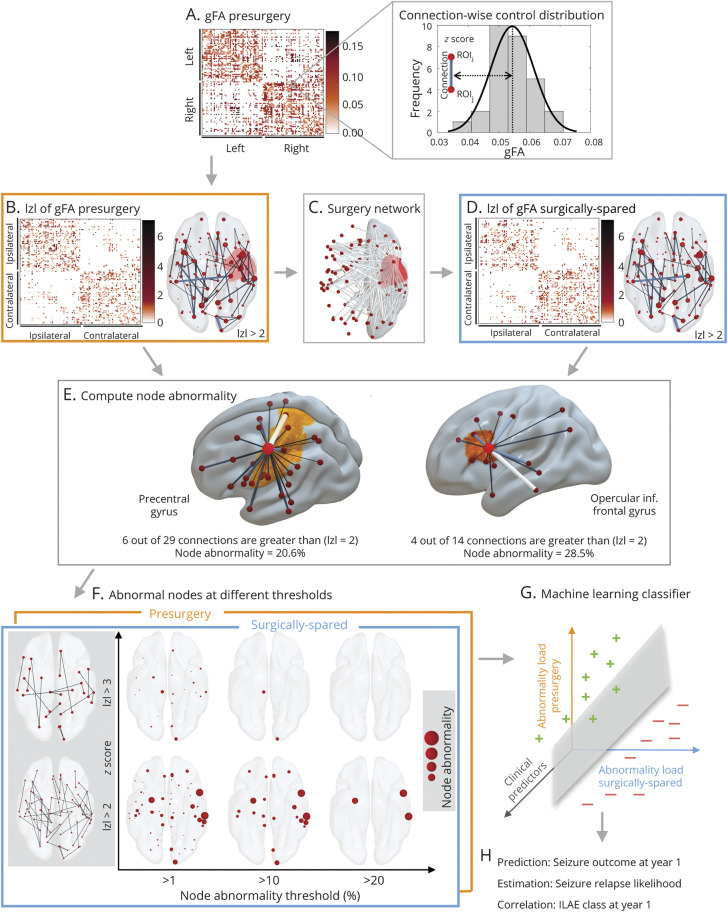
Overall Pipeline Presurgical generalized fractional anisotropy (gFA) network architecture for each patient in panel (A) is inferred, and connections between regions of interest are standardized against a control distribution (illustrated for an example connection in the right of panel [A]) to obtain a *z* score–transformed network in panel (B). The connections affected by the surgery shown in surgery network in panel (C) are removed to obtain surgically spared network in panel (D). (E) Concept of node abnormality for 2 example nodes. By normalizing the number of abnormal links to a node with its degree, the heterogeneity in the degree of network nodes is accounted for. An h-degree node can be less abnormal compared to a low-degree node depending on the number of abnormal connections. (F) Different thresholds required for the computation of node abnormality are shown for surgically spared network (blue panel front) and the presurgery network (orange panel back). The *z* score at which a link is considered abnormal is on the y-axis, and the cutoff at which a node is considered abnormal is shown on the x-axis. (G and H) Abnormality loads in presurgery and surgically spared networks are incorporated into a machine learning classifier along with the clinical predictors to predict seizure outcomes at year 1 both in terms of binary seizure-free (plus in green) vs not seizure-free (minus in red) outcome and in terms of the probability with which each patient was classified as not seizure-free. These probabilities correlated with severity of seizure outcome (i.e., International League Against Epilepsy [ILAE] class) at year 1 and associated with the seizure relapse in 5 years. We called these probabilities predicted seizure relapse likelihood. |z| = z-score

We computed node abnormality on networks based on the mean generalized fractional anisotropy (gFA) property of dMRI streamlines.^35^ The presurgery networks were standardized patient-specifically against controls as follows: for each connection present between ROIs i and j in a patient, the connection distribution was obtained from the equivalent connection between ROIs i and j from the control networks. The *z* score for that connection was calculated as the number of SDs away from the mean, where the SD and mean were obtained from the control distribution. Networks inferred from deterministic tractography are sparse, so we *z* scored only those connections in patients for which an equivalent connection existed in at least 10 (≈35%) controls.^36^ This is depicted in figure 2A.

After obtaining the presurgery *z*-scored gFA network, we removed the connections present in the surgery-affected network to obtain the surgically spared *z*-transformed gFA network. High 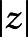 indicates high deviation from normality. Thus, the presurgery network maps the abnormal links present before the surgery, and the surgically spared network maps the abnormal links that would remain unaffected immediately after the surgery. This is illustrated in figure 2, B through D.

To study how different regions (nodes) are affected in these networks, we computed node abnormalities (figure 2E) in the presurgery and surgically spared networks by counting the number of abnormal links to each node. We normalized the number of abnormal links to a node by its degree in the presurgical network, thus expressing node abnormality in percentage terms.

Quantification of node abnormality load raises 2 questions. First, what is the definition of an abnormal connection? Second, when is a node considered abnormal? The former is essential for the application of a threshold on the abnormality network to count the number of abnormal links at each node. For the latter, another threshold is needed to define beyond what percentage level a node should be considered abnormal. We therefore varied the *z*-score threshold from 2.1 to 4.5 in increments of 0.1 and the percentage abnormality threshold from 1% to 50% in increments of 1%. At each point on this 2-dimensional grid, we computed how many nodes were abnormal in presurgery and surgically spared networks. We call this the abnormality load, the total number of abnormal nodes identified at any given pair of thresholds. This is illustrated in figure 2F for 6 example threshold pairs. Finally, having quantified the abnormality load for each patient, we assessed its discriminatory ability in predicting seizure outcome at year 1 and seizure relapse in 5 years.

### Quantifying the Change in Abnormality Load After ATLR

To investigate the effect of surgical treatment on reduction of abnormalities, we compared the difference in abnormality load between presurgery and surgically spared networks. The ROIs in the left and right hemispheres of patients were expressed as ipsilateral or contralateral to surgery. Then, we categorized each ROI into 6 ipsilateral and 6 contralateral areas, i.e., temporal, subcortical, parietal, occipital, frontal, and cingulate cortices. In each area, we determined the number of abnormal nodes in the presurgery and surgically spared networks patient-specifically. Then, we averaged the number of abnormal nodes in each area for seizure-free (ILAE 1) and not seizure-free (ILAE 3–5) groups of patients. Finally, by computing the proportion of abnormal nodes in every area (i.e., ratio of mean abnormal ROIs to the total number of ROIs in each area) for the presurgery and surgically spared network, we noted the change due to the surgery in all patients.

### Predictive Model Design for Generalizability Assessment

To predict the patient-specific probability of seizure relapse based on preoperative clinical data (table 1 and table S1, doi.org/10.5061/dryad.vx0k6djnv) and presurgical and surgically spared node abnormality, we applied support vector machine (SVM) learning algorithm.^37,38^ The outcome values for SVMs were the seizure outcome at year 1, with ILAE 1 labeled as seizure-free outcome and ILAE 3 to 5 labeled as not seizure-free outcome. We incorporated a linear kernel in SVM because this enabled a direct interpretation of weights as relative feature importance. We performed nested-cross-validation in which the data were split into 3 folds: training, validation, and testing. During training, SVM learned from the data in the training fold only; it did not see any data in the validation or test fold. Node abnormality measures were computed afresh for each patient in the training fold, and the most discriminatory (highest area under the curve [AUC]) threshold pairs across patients in the training fold were noted. A trained SVM learned to weigh the 15 preoperative attributes in the order of their relative importance to maximize the training accuracy. We avoided overfitting by incorporating a regularization parameter in SVM. The regularization parameter was optimized on the validation fold, after which the SVM was tested for generalizability on the unseen data in the test fold. This is akin to a new incoming patient (pseudoprospective), for which the learning algorithm has been trained and optimized on existing/past patient records.

During the training phase, the SVM draws a linear hyperplane to separate patients who were seizure-free (ILAE 1) from those who were not seizure-free (ILAE 3–5) at year 1. The features of the test patient are then tested on this hyperplane, and depending on how confidently the SVM places this patient in the not seizure-free group, a probability is assigned. We refer to these probabilities as the likelihood of seizure relapse because a high probability indicates a predicted propensity toward a not seizure-free outcome. By implementing a leave-one-out scheme, we measured (1) the net generalizability of the learning algorithm (quantified by AUC, accuracy, sensitivity, and specificity), (2) how correlated the predicted probabilities of seizure relapse are with severity of seizure outcomes (i.e., ILAE class at 1 year), and (3) the association between actual relapse in 5 years and the predicted probabilities of seizure relapse.

Ranking features indicated metrics that were important for prediction performance as opposed to those that may be confounding. Therefore, to identify the combination of most informative features, we removed the least informative feature and repeated the above process until a single feature remained.^38,39^ More details on data splits and nested cross-validation are provided in supplementary materials, doi.org/10.5061/dryad.vx0k6djnv.

### Statistical Analysis

To investigate whether a greater number of abnormal nodes are associated with not seizure-free (ILAE 3–5) outcomes, we applied the nonparametric Wilcoxon rank-sum test. A one-tailed *p* value was computed with the ranksum function in MATLAB (MathWorks, Inc, Natick, MA) incorporating the exact method. Results are declared significant for *p* < 0.05. We further applied Benjamini-Hochberg false discovery rate correction^40^ at a significance level of 5%. Effect size between groups was computed with the Cohen *d* score, and the correlation coefficients between likelihood of seizure relapse and the severity of seizure outcome were determined with the Spearman rank order. We computed 95% bootstrap confidence intervals (CIs) of effect size, AUC, and median using a bias-corrected and accelerated percentile method from 10,000 bootstrap resamples with replacement. Our study provides Class II evidence that node abnormality before and expected to remain after surgery predicts postsurgical seizure recurrence.

### Data Availability

To enable reproducibility, we will make available all the anonymized presurgery and surgically spared brain networks of 51 patients, networks of 29 controls, codes for node abnormality computations, and all the trained machine learning models on the data presented. Supplementary data, text, and figures are available at Dryad (doi.org/10.5061/dryad.vx0k6djnv.

## Results

The results are organized into 3 parts. First, we assessed whether patients with a greater number of abnormal nodes (i.e., a higher abnormality load) are predisposed to ILAE 3 to 5 (poorer) seizure outcome after surgery. Outcomes are initially measured at 12 months and then at later years. Second, we investigated the effect of surgery in reducing the node abnormality load between the seizure-free and not seizure-free groups. Third, we determined the generalizability of node abnormality measure, if it is to be incorporated in a clinical setting alongside other clinical attributes, to estimate the chances of seizure recurrence for new patients.

### Abnormality Load Corresponds With Year 1 Surgical Outcome and Later-Year Seizure Relapse

**Figure 3:**
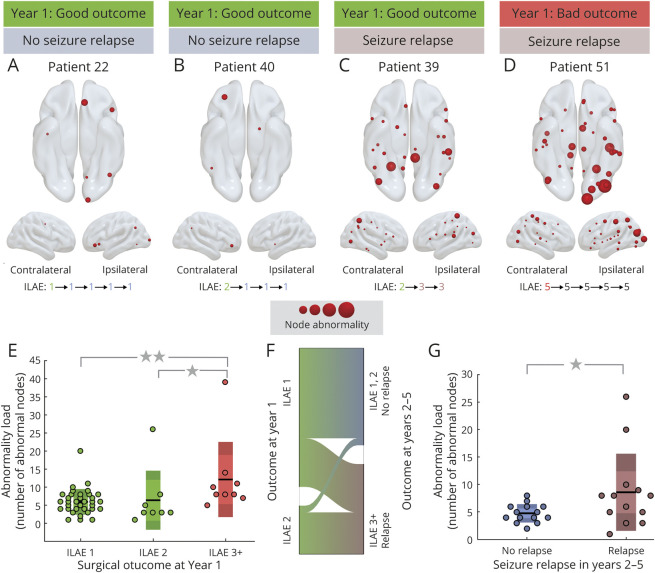
Association Between the Number of Abnormal Nodes In Surgically Spared Network With Year 1 Surgical Outcome and Relapse (A–D) Four patients are shown with their year 1 surgical outcome and relapse information. (A) and (B) Lower abnormality load in patients with International League Against Epilepsy (ILAE) 1 and ILAE 2 outcomes, respectively, with no relapse. (C) Patient with many abnormal nodes remaining yet having an ILAE 2 outcome at year 1 but relapsing subsequently. (D) Large number of abnormal nodes remaining in a patient who was never seizure-free in 5 years. (E) Significantly more abnormal nodes remained in ILAE 3+ patients compared to ILAE 1 and ILAE 2 patients. Statistical estimates: ILAE 1 (n = 34) median 6 (95% confidence interval [CI] 5–7.5); ILAE 2 (n = 8) median 3 (95% CI 2–5.5); ILAE 3+ median 8 (95% CI 5–10); *p*(ILAE 1 vs ILAE 3+) = 0.005; d(ILAE 1 vs ILAE 3+) = 1.11 (95% CI 0.42–2.2); p(ILAE 2 vs ILAE 3+) = 0.01; d(ILAE 2 vs ILAE 3+) = 0.61 (95% CI −0.92 to 2.04). (F) Alluvial flow diagram showing proportion of relapsed patients with ILAE 1 or ILAE 2 at year 1. (G) In ILAE 1 to 2 patients, those who relapsed had significantly more abnormal nodes in the surgically spared network. Statistical estimates: no relapse (n = 14) median 4.5 (95% CI 4–6); relapse (n = 13) median 8 (95% CI 5–15); *p* = 0.04; d = 0.77 (95% CI −0.01 to 1.31).

We investigated the abnormality load in surgically spared and presurgical networks. Figure 3, A through D illustrates abnormal nodes in the surgically spared networks for 4 patients. The patients in figure 3, A and B were seizure-free (ILAE 1) and had auras (ILAE 2) at 1 year after surgery and did not relapse subsequently; they had a relatively low node abnormality load. The patient in figure 3C initially had auras only (ILAE 2) at 1 year after surgery but later relapsed; this patient showed a higher abnormality load. The patient in figure 3D, with the highest abnormality load, had the worst surgical outcome of ILAE 5 at 1 year, which persisted on follow-up. In these 4 cases, a greater abnormality load was associated with worse outcomes at year 1 and with seizure relapse at later years.

Figure 3E shows the node abnormality load in surgically spared network for the entire patient cohort. Patients who were not seizure-free (ILAE 3+) at 1 year after surgery had a significantly higher abnormality load than patients who were seizure-free (*p* = 0.005, d = 1.11 [95% CI 0.42–2.2] between ILAE 1 and ILAE 3–5; and *p* = 0.01, d = 0.61 [95% CI −0.92 to 2.05] between ILAE 2 and ILAE 3–5). Here, we chose to analyze ILAE 2 as a separate group because clinical data (table 1 and table S1, doi.org/10.5061/dryad.vx0k6djnv) suggest that these patients, albeit free from disabling seizures at year 1, have a greater propensity to relapse in later years.^41^ Studying only the subset of patients who were initially seizure-free (i.e., ILAE 1–2) at 1 year (figure 3, F and G), patients who relapsed had a higher abnormality load than the patients who did not relapse (*p* = 0.04; d = 0.77 [95% CI −0.01 to 1.31]).

Node abnormality in figure 3, computed from the surgically spared network, was defined as the nodes with at least 10% of abnormal (*z* > 2.8) connections. At this choice of thresholds, the discrimination (AUC) between the seizure-free and not seizure-free groups was the highest. Comparable results are found for other threshold values (supplementary figure S1, doi.org/10.5061/dryad.vx0k6djnv) and with an alternative network parcellation (supplementary figure S4). Thus, the discriminatory ability of the node abnormality load measure is consistent across the choice of threshold or the choice of parcellation scheme.

We found similar results in the presurgery networks. Patients at ILAE 3 to 5 had significantly more abnormal nodes than patients at ILAE 1. However, the size of this effect was less pronounced than in the surgically spared networks, with relatively poorer discriminatory ability (*p* = 0.03; d = 0.78 [95% CI 0.04–2.1]) (see, supplementary figures S1 and S2, doi.org/10.5061/dryad.vx0k6djnv). Therefore, our findings suggest that the surgically spared network, which is the surgically informed subnetwork of the presurgery network, is more discriminatory in identifying seizure-free from not seizure-free patients.

### Surgery-Related Effect on Reducing Abnormality Load

**Figure 4:**
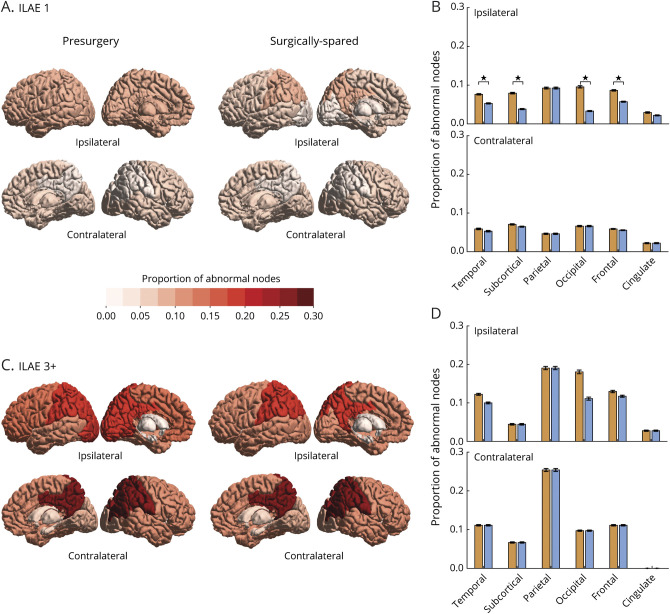
Effect of Surgery in Reducing Node Abnormality Is More Widespread in the Seizure-Free Group at Year 1 (A) Proportion of abnormal nodes computed for presurgery and surgically spared networks are color-coded for 6 ipsilateral and contralateral brain areas in the seizure-free (International League Against Epilepsy [ILAE] 1) group. (B) Bar plot shows the drop in the proportion of abnormal nodes in surgically spared network compared to presurgery network. Error bars represent standard error of the proportion of abnormal nodes in each area. Significant reductions in ipsilateral temporal (*t* = 3.19, mean 0.02 [95% confidence interval (CI) 0.01–0.04], *p* = 0.003), ipsilateral subcortical (*t* = 4.31, mean 0.04 [95% CI 0.02–0.06], *p* < 0.001), ipsilateral occipital (*t* = 2.94, mean 0.06 [95% CI 0.02–0.11], *p* = 0.006), and ipsilateral frontal (*t* = 3.21, mean 0.03 [95% CI 0.01–0.05], *p* = 0.002) areas are indicated by stars. (C) Different brain areas in presurgery and surgically spared network are color-coded according to the proportion of abnormal nodes in the not seizure-free group (ILAE 3+). (D) Corresponding bar plot shows the drop in the proportion of abnormal nodes in surgically spared network compared to presurgery network. None of the ipsilateral or contralateral areas showed a significant reduction in the proportion of abnormal nodes.

How much effect does surgery have on reducing the abnormality load? We investigated the differences between the surgically spared and presurgery networks in terms of their abnormality load and whether the projected change in abnormality load due to surgery was greater and more widespread in one outcome group compared to another. The proportions of abnormal nodes in different brain areas for ILAE 1 (seizure-free) and ILAE 3 to 6 (not seizure-free) groups are shown in figure 4, with the intermediate patients at ILAE 2 shown in supplementary figure S5, doi.org/10.5061/dryad.vx0k6djnv.

In terms of the spatial extent of surgery, the expected reduction in the proportion of abnormal nodes was more widespread in the seizure-free group than in the not seizure-free group. The ILAE 1 group had a significant drop in the proportion of abnormal nodes in the surgically spared network compared to the presurgical network in 4 ipsilateral areas: temporal, subcortical, occipital, and frontal (figure 4, A and B). In the ILAE 3 to 5 group, however, the drop in the proportion of abnormal nodes was not significant in any of the ipsilateral or contralateral areas (figure 4, C and D). A similar surgery-related effect was found for node abnormality computed at different threshold values (supplementary figure S3, doi.org/10.5061/dryad.vx0k6djnv).

In terms of reduction in the amount of abnormality load, patients at ILAE 1 had larger proportional reductions than patients at ILAE 3 to 5 (*p* = 0.01; d = 0.81 [95% CI 0.2–1.4]); however, their absolute reduction did not differ significantly (*p* = 0.14; d = 0.42 [95% CI −0.25 to 0.84], see supplementary figure S5, doi.org/10.5061/dryad.vx0k6djnv). Thus, we suggest that the TLE surgery causes a greater and widespread reduction in abnormality load in the seizure-free group than in the not seizure-free group.

### Personalized Prediction of 12-Month Seizure Freedom Additionally Suggests ILAE Class and Relapse at Later Years

We assessed the generalizability of the abnormality measure when used alongside other clinical attributes to predict patient-specific chances of poorer outcomes. Implementing nested cross-validation, we built machine learning models that classified new unseen (test) patients as belonging to either the ILAE 1 or the ILAE 3 to 5 group at 12 months. Our rationale for omitting patients at ILAE 2 from the training phase of the model was that, because they have a propensity to develop seizures in the later years, they therefore lie on a spectrum in between the seizure-free group and the not seizure-free group. The model also scored each patient with a probability of belonging to either of the classes. Notably, the models were blinded to 3 aspects of the data: (1) all patients at ILAE 2; (2) ILAE classification 3, 4, and 5 (the model simply sees these as poor outcome); and (3) outcomes at later years.

**Figure 5:**
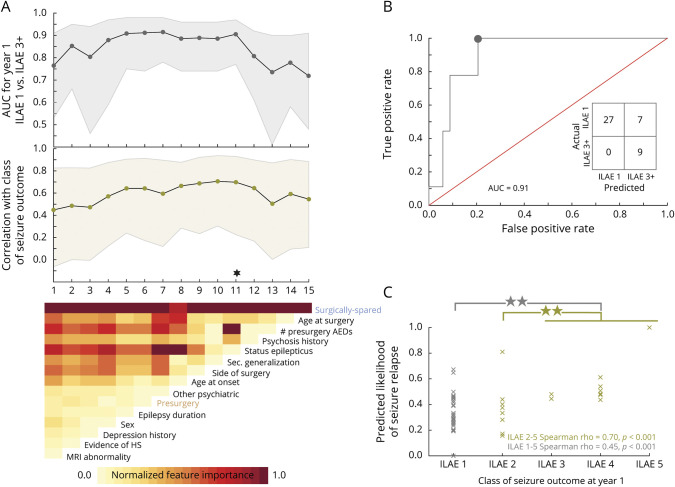
Prediction of Seizure Outcomes at Year 1 (A) Areas under the curve (AUCs) of support vector machines (SVMs) that predicted seizure-free (International League Against Epilepsy [ILAE] 1) and not seizure-free (ILAE 3+) outcomes at 1 year after surgery are plotted in black with 95% confidence interval (CI) shaded. Blinded to the exact ILAE categories, the model predicted 12-month likelihood of seizure relapse for each patient. The Spearman rank correlation between likelihood of seizure relapse and the severity of surgical outcomes (ILAE class) at year 1 is plotted in green with 95% CI shaded. The lower panel of (A) shows the relative feature importance of each SVM on a normalized scale between 0 and 1. The leftmost SVM, plotted at x = 1, incorporated all 15 features (13 clinical, node abnormality in presurgery and surgically spared networks) to predict seizure-free (ILAE 1) and not seizure-free (ILAE 3+) outcomes at 1 year after surgery. Among all features, the relative importance of surgically spared node abnormality was the highest, whereas the relative importance of MRI abnormality was the least. Therefore, in the next iteration at x = 2, a new SVM was retrained using the 14 features, after removal of the MRI abnormality feature. This stepwise removal of metrics was continued until only a single metric (surgically spared node abnormality) remained. (B) Receiver operating characteristics curve is plotted at an example combination of features that yielded highest classification performance (AUC = 0.91 [95% CI 0.77–0.97], specificity = 1, sensitivity = 0.79, accuracy = 0.84). (C) At the same example point, the correlation (Spearman ρ = 0.70 [95% CI 0.25–0.93], *p* < 0.001) between the predicted likelihood of seizure relapse and the severity of seizure outcome at year 1 is shown. The predicted likelihood of seizure relapse was significantly different (*p* = 0.003, d = 0.96 [95% CI −0.29 to 2.1]) between ILAE 2 (n = 8, median 0.35 [95% CI 0.17–0.43]) and ILAE 3 to 5 patients combined (n = 9, median 0.48 [95% CI 0.44–0.61]). AED = antiepileptic drug.; HS = Hippocampal sclerosis.

We incorporated up to 15 features in the model: 13 clinical attributes, presurgical abnormality load, and surgically spared abnormality load. These features describe the presurgical attributes of patients, and we evaluated them on the basis of their combined ability in accurately predicting surgical outcomes at 1 year. However, some features may be less informative than others in predicting surgical outcomes; including less informative features causes a drop in the prediction performance. Therefore, by implementing stepwise removal of less informative features, we obtained combinations of preoperative features that identified patients with poor seizure outcome at 1 year after surgery in 100% cases (i.e., specificity). The AUC at every step of feature elimination is plotted in figure 5A and magnified at 1 example point (marked with a star) in figure 5B with the corresponding confusion matrix shown in the inset. Average prediction performance across all stepwise feature removals was robust (AUC 0.84 ± 0.07, accuracy 0.79 ± 0.05, specificity 0.89 ± 0.09, sensitivity 0.77 ± 0.06). Supplementary table 2, doi.org/10.5061/dryad.vx0k6djnv, tabulates these prediction metrics in classifying seizure-free and not seizure-free outcomes at every step. The lower panel in figure 5A maps feature importance after each iteration of feature removal. The node abnormality in the surgically spared network stood out as the most informative feature; it was >1.5 SD away from the next most important features: age at surgery and number of antiepileptic drugs taken before surgery. Thus, including the abnormality measures to characterize presurgical attributes of patients with intractable TLE led to a high and robust classification performance in predicting surgical outcomes at 1 year after surgery.

We next analyzed the scores/probabilities assigned by the model to each patient to have a not seizure-free surgical outcome. Larger probabilities indicated a greater predicted likelihood of postoperative seizure at year 1 (i.e., the ILAE 3+ group). Because the model was trained only on binary ILAE 1 and ILAE 3–5 outcomes, it was blinded to the spectrum of ILAE class data. We found that, despite being blinded to such information, the predicted likelihood of seizure relapse was positively correlated with ILAE surgical outcome scale at year 1 (figure 5C). This positive association is consistent, even for the model trained with only the surgically spared node abnormality feature (figure 5A). Spearman ρ values are plotted at each step-wise removal of features in figure 5A and magnified for an example point in figure 5C. To confirm this result, we applied robust regression to obtain the regression slope and tested the significance of the steepness of the regression slope using a permutation test (1,000 permutations, *p* = 0.004 in supplementary figure S6, doi.org/10.5061/dryad.vx0k6djnv). Therefore, our result shows that the presurgical clinical profile of patients, when assessed along with the abnormality measures, can inform about the ILAE class of seizure outcomes that a patient would expect after surgery at 1 year.

**Figure 6:**
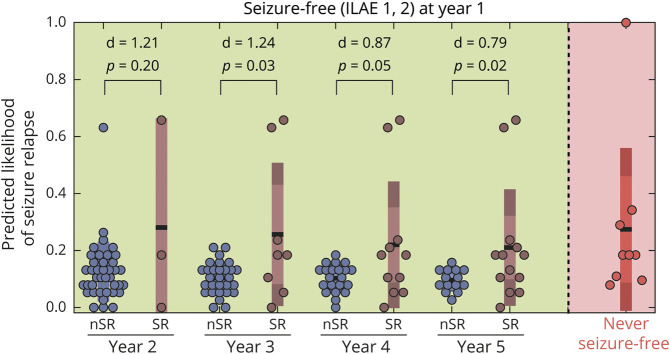
Predicted Likelihood of Seizure Relapse at 1 Year Was Higher in Patients Who Had Seizure Relapse at Later Years The predicted 12-month likelihood of seizure relapse was estimated from the support vector machine model trained with only the surgically spared node abnormality feature. The likelihood of seizure relapse for patients who were never seizure-free (i.e., ILAE 3–5 at year 1) is shown in red. Among the patients who were initially seizure-free (i.e., ILAE 1 or ILAE 2 at year 1), higher likelihood of seizure relapse was predicted for those who had a subsequent relapse. This is despite the model being blinded to the outcomes at later years. Year 2 statistical estimates: no relapse (n = 39) median 0.10 (95% confidence interval [CI] 0.08–0.13); relapse (n = 3) median 0.18 (95% CI 0–0.66); *p* = 0.20; d = 1.21 (95% CI −0.78 to 9.08). Year 3 statistical estimates: no relapse (n = 29) median 0.11 (95% CI 0.08–0.13); relapse (n = 8) median 0.18 (95% CI 0.05–0.63); *p* = 0.03; d = 1.24 (95% CI −0.19 to 2.84). Year 4 statistical estimates: no relapse (n = 21) median 0.11 (95% CI 0.08–0.13); relapse (n = 11) median 0.18 (95% CI 0.05–0.41); *p* = 0.05; d = 0.87 (95% CI −0.16 to 1.7). Year 5 statistical estimates: no relapse (n = 14) median 0.09 (95% CI 0.08–0.13); relapse (n = 13) median 0.18 (95% CI 0.11–0.24); *p* = 0.02; d = 0.79 (95% CI 0.12–1.3). nSR = No seizure relapse; SR = Seizure relapse.

How informative are the presurgical features in predicting seizure recurrences in the long term? We analyzed this by checking the association between the predicted likelihood of seizure relapse and the actual relapse data for patients who were seizure-free (ILAE 1–2) at year 1. Patients who were not seizure-free at year one (ILAE 3–5) were not included in the relapse category. We found no association with seizure relapse when the presurgical features of patients were characterized using a combination of clinical and network abnormality measures (supplementary figure S7, doi.org/10.5061/dryad.vx0k6djnv). However, significant association with relapse was present at years 3, 4, and 5 when the presurgical features of patients were characterized using only the abnormality load in surgically spared network (figure 6). The mechanism of long-term seizure recurrence may be different from short-term recurrence, and presurgical clinical attributes may be less informative. The association we found between abnormality load expected to be present in a patient after surgery and seizure recurrence motivates more investigation.

In summary, we achieved excellent performance in predicting seizure outcomes at 1 year when patients with intractable TLE were assessed according to abnormality measures and clinical attributes. This combined presurgical profiling of patient attributes was also informative about the grades of seizure outcomes (ILAE class) at year 1. Beyond the first year after surgery, node abnormality in the surgically spared networks also suggested an increased risk of seizure relapses in those patients who were initially free of disabling seizures at year 1.

## Discussion

We investigated the association of surgical outcomes and relapse with the abnormality load computed in a whole-brain presurgical network and surgically spared subnetwork. Patients were more likely to have a poorer seizure outcome at 1 year after surgery or a seizure relapse in 5 years if more abnormal nodes were present in the surgically spared network. Investigating the spatial effect of surgery on abnormality load, we found that the seizure-free group of patients had a more widespread reduction of abnormal nodes. We found that the abnormality load in presurgery and surgically spared networks, combined with clinical attributes of patients, generalized to predict not seizure-free outcome (between ILAE 1 and ILAE 3+) at 12 months after surgery with 100% specificity and an AUC of 0.91. With this combined characterization of patient attributes, we predicted the likelihood of seizure relapse patient-specifically, which was correlated with the ILAE class and hence informative of the seizure outcome expected at 12 months after surgery. Finally, we showed that node abnormality located in the surgically spared networks may be a marker that identifies patients who were initially seizure-free but would relapse after the first year of surgery and up to 5 years.

A recent study on a different dataset with different imaging protocols investigated network abnormality as a personalized predictor of surgical outcomes.^13^ In that study, presurgery networks were constructed on the basis of the number of streamlines connecting different regions. Similar to our study, connections between ROIs were normalized (*z* transformed) against a control distribution. The similarity between our results suggests that (1) normalized patient networks using a local control distribution may enable reproducibility, comparison, and possibly grouping of patients between sites and (2) noninvasive personalized network biomarkers for predicting the likelihood of specific postsurgery outcomes in TLE are possible. We further showed the benefit of incorporating the information about the location of surgery to predict the surgical outcome.

The current standard for individualized prediction of surgical outcome primarily relies on clinical variables.^42^ However, a recent review discussed discordant findings between different studies; features found predictive of seizure outcome in some studies are not predictive in others.^30^ Encouragingly, another study estimated the probability of seizure freedom using combinations of up to 27 clinical variables on a mixed cohort of patients with TLE and extratemporal TLE.^9^ Our findings indicated that combining clinical variables with brain connectome–derived features such as abnormality load in presurgery and surgically spared networks can improve prediction of surgical outcomes in the short term. Particularly for long-term predictions, the abnormalities in the surgically spared networks, which are expected to remain after surgery, may be a more reliable measure because they associate with relapses. It is not surprising that long-term seizure relapse is not specifically related to parameters predicting short-term outcome well because these 2 responses may have very different mechanisms. Short-term failure may be caused by an incomplete resection at the time of surgery, while long-term relapse may be caused by changes in the networks over time after surgery or the development of another epileptogenic zone.^43^ Hence, we propose to combine our node abnormality measure with clinical variables in a large mixed-cohort patient^9^ population to improve estimation of the probability of seizure freedom/relapse after surgery. We suggest that investigating the abnormality load in the surgically spared network in specific lobes may reveal a stronger relationship to long-term seizure recurrence.

In combining multivariate data, machine learning techniques delineate, rank, and fit input features of the training set to draw a decision boundary in a high dimensional space that maximizes prediction.^13,14,20,21,44^ While a binary classification of seizure-free and not seizure-free outcomes at 12 months is important, predicting long-term trajectories of seizure freedom is also crucial to inform clinical management decisions. In our study, the classifier not only predicted the surgical outcome at 1 year but also predicted the likelihood of seizure relapse. This additional information may be clinically useful for advising patients about their chances of poor outcome after surgery beyond the first 12 months.

The outcome of epilepsy surgery will depend not only on the brain network before the surgery but also on the location and extent of surgery.^21,45^ Here, we retrospectively included the information of surgery by drawing a resection mask and inferring an expected surgically spared network. We showed that this information improves the prediction performance more so than just the presurgery networks, which are naive to surgical information. A limitation of our work is that we are only inferring the expected postoperative network rather than deriving it from postoperative dMRI data.^43,46^ However, an analysis using actual postoperative dMRI data would have only very limited value in terms of improving the preoperative decision-making because the outcome could be seen only after the surgery has been performed. In contrast, our approach can be used before the actual surgery to evaluate likelihood of success. A prospective application would involve drawing a resection mask for an intended surgery on the sMRI of a patient acquired before surgery.^21^ We envisage a software tool with which multiple standard operations or tailored resections could be drawn and their impact on abnormality load compared. Such a tool could then be used to prospectively guide decision-making regarding personalized resection strategies.

With regard to the extent of surgical resection, it has been shown that the amount of tissue resected does not necessarily relate to improved surgical outcome.^47^ What is included in the resection, however, may have a significant influence on outcome.^48,49^ The question arises: will a tailored resection, designed to reduce the number of abnormal nodes, lead to a better outcome? While more investigations are needed to confirm this hypothesis, we found that the seizure-free patient group had a more widespread reduction of abnormality load due to surgery. Simulated computer models may facilitate a more detailed analysis to investigate alternative surgical strategies in a personalized manner.^19^

Our findings must be interpreted in the context of some caveats. Node abnormality may be representative of (1) network reorganization in response to seizures, (2) neurodegenerative process due to seizures, (3) structures facilitating seizures, or combinations of 1 through 3. In our study we could not disentangle these aspects with respect to node abnormality. We did not detect any significant correlation between clinical variables and node abnormalities. Although our sample size is reasonably large,^13,14,16,20^ it is not of the size of typical epidemiologic studies. Neural architecture depends on several participant-specific factors, including language dominance, handedness, and other physiologic variables. These relationships may further influence the node abnormality measure. Thus, our results should motivate a larger study to test its generalizability, ideally across multiple sites. Finally, we highlight, on the basis of the presurgical and surgically spared networks and clinical variables, the chances of at least 1 relapse in 5 years. However, the trajectory of seizure remission and relapse is more complicated. Patients may have repeated remissions and relapses due to drug effects, environmental factors, or other causes.

We have shown evidence of node abnormality being an important noninvasive marker of surgical outcome and its severity at 1 year after surgery. Node abnormality may also be related to likelihood of seizure relapse in the long-term. We demonstrate improvement in prediction performance when including surgery information with the presurgery network and clinical data. We believe this to be an important step toward complementing clinical decision-making on patient and surgery selection for intractable TLE and for patient counseling on the risks of seizure severity expected after surgery.

## Glossary

AUC: area under the curve
CI: confidence interval
dMRI: diffusion-weighted MRI
gFA: generalized fractional anisotropy
ILAE: International League Against Epilepsy
ROI: region of interest
sMRI: structural MRI
SVM: support vector machine
TLE: temporal lobe epilepsy
